# CDCA7 promotes chemoresistance of drug-tolerant persister cells in breast cancer by upregulating the expression of autophagy-related protein genes

**DOI:** 10.3389/fimmu.2026.1782047

**Published:** 2026-03-11

**Authors:** Jin Wu, Zhaoyu Wang, Juan Liu, Qinghua Ma, Sisi Li, Rong Zhou, Jingya Miao, Qingqiu Chen, Jun Jiang, Wei Liu, Peng Tang

**Affiliations:** 1 Department of Breast and Thyroid Surgery, Southwest Hospital, the First Affiliated Hospital of the Army Medical University, Chongqing, China; 2 Institute of Pathology and Southwest Cancer Center, Southwest Hospital, the First Affiliated Hospital of the Army Medical University, Chongqing, China; 3 The Biobank of Southwest Hospital, the First Affiliated Hospital of the Army Medical University, Chongqing, China

**Keywords:** autophagy, breast cancer, CDCA7, drug-tolerant persister state, transcriptional regulation

## Abstract

**Background:**

Chemotherapy resistance is the main obstacle to breast cancer recurrence, metastasis, and mortality. Drug-tolerant persister (DTP) cells are a novel type of target cell associated with tumor resistance, and autophagy is a key factor in maintaining the survival of tumor DTP cells. However, it is unclear whether the activation of autophagy in breast cancer DTP cells is related to their overexpression of the transcriptional regulatory factor CDCA7.

**Methods:**

We analyzed CDCA7 expression using public datasets and clinical samples and established breast cancer cell lines with CDCA7 overexpression and knockdown to assess the role of CDCA7 in breast cancer. Autophagy was assessed via electron microscopy, mRFP-GFP-LC3 imaging, and immunoblotting. Mechanistic studies employed ChIP-seq, dual-luciferase assays, and site-directed mutagenesis. Functional assays measured chemosensitivity (CCK-8), migration/invasion (scratch/Transwell), and *in vivo* tumorigenicity (mouse xenograft).

**Results:**

CDCA7 was significantly upregulated in breast cancer DTP cells. Overexpression of CDCA7 in breast cancer cells significantly enhanced autophagy-related biological processes and molecular functions. Through ChIP-seq and targeted knockout experiments, we identified the binding sites of CDCA7 on the autophagy-related protein genes *ULK1*, *ATG2A*, and *ATG3*. Using transmission electron microscopy and mRFP/mCherry-GFP-LC3B tandem fluorescent tagging, we observed that *CDCA7* knockdown significantly reduced the number of autolysosomes in breast cancer DTP cells and markedly inhibited autophagic flux. Moreover, *CDCA7* knockdown not only decreased drug resistance in breast cancer cells but also reduced metastasis, invasion, and tumorigenic ability *in vivo*, ultimately prolonging the survival of tumor-bearing mice.

**Conclusion:**

CDCA7 drives breast cancer chemoresistance by transcriptionally activating a pro-survival autophagy program in DTP cells, nominating it as a promising therapeutic target.

## Introduction

Female breast cancer is one of the tumors with the highest incidence and mortality rates worldwide (1). Chemotherapy is an important method for treating breast cancer, especially for advanced luminal breast cancer and triple-negative breast cancer (2). However, tumor cells develop adaptations through various mechanisms, such as activating drug efflux pumps, enhancing DNA repair mechanisms, and altering the tumor microenvironment, leading to chemotherapy failure and significantly limiting the clinical efficacy of breast cancer treatment (3, 4). The current main strategies to overcome chemoresistance in breast cancer include combination therapies (such as inhibitors targeting the PI3K/AKT/mTOR pathway and anti-angiogenic drugs, immune checkpoint inhibitors, and IL-2 cytokine therapy) and chemosensitizers (such as pazopanib, 2-deoxy-D-glucose, isoliquiritigenin, and metformin) (5–8). However, clinical application still faces challenges such as poor patient compliance, high treatment costs, long-term toxicity, and a high risk of tumor recurrence and metastasis (2, 4). Therefore, it is crucial to investigate the potential mechanisms of chemotherapy resistance in breast cancer and to develop effective targeted therapies.

Recently, drug-tolerant persister (DTP) state cells, which exhibit significant phenotypic differences from tumor stem cells and drug-resistant tumor cells, have attracted widespread attention (9, 10). Under the stress of chemotherapy or targeted therapy, tumor cells can enter the DTP state randomly and reversibly, displaying characteristics such as cell cycle arrest, activation of autophagy, increased epithelial-mesenchymal transition, and metabolic reprogramming (9). Among these, active autophagy serves as a crucial material basis for the survival of DTP cells and represents a potential new therapeutic target for directly killing DTP cells (11).

Autophagy plays a dual role in the drug resistance process of tumor cells (12, 13). On one hand, autophagy provides energy for cellular metabolism and the renewal of certain organelles (14). On the other hand, when cells experience excessive stress, such as endoplasmic reticulum stress, oxidative stress, or starvation, they can induce apoptosis through autophagic ‘self-destruction’ (15–17). Whether autophagy promotes cell survival or death depends on the type of tumor cells and the specific resistance mechanisms involved. In some drug-resistant tumor cells, the expression levels of apoptotic genes are reduced, and in this context, adaptive autophagy can increase the drug resistance of these cells. However, under certain specific conditions, excessive autophagy can eliminate some anti-apoptotic signaling pathways, thereby increasing the sensitivity of resistant cells to chemotherapy (12). Revealing the mechanisms regulating autophagy in breast cancer DTP cells will help deepen the understanding of the molecular mechanisms of breast cancer drug resistance and provide experimental evidence for overcoming breast cancer drug resistance by breaking the DTP state through autophagy inhibition.

Cell division cycle-associated protein 7 (CDCA7) is a transcriptional regulator that mediates c-Myc and E2F1-regulated cell cycle progression and DNA damage repair, playing a crucial role in cell growth, survival, metabolism, embryonic development, and immune responses (18, 19). Abnormal expression of the CDCA7 gene is associated with the occurrence and progression of various tumors. CDCA7 is abnormally overexpressed in multiple cancers, including breast cancer, lung cancer, and colorectal cancer (20–22). Overexpression of CDCA7 may promote tumor cell resistance, survival, and metastatic invasion, whereas its low expression may increase apoptosis (23, 24). The precise role of CDCA7 in the sustained drug-resistant state of breast cancer remains unclear.

Therefore, this study aimed to investigate the expression levels and functional significance of CDCA7 in breast cancer DTP cells. CDCA7 was highly expressed across various subtypes of breast cancer. It was closely associated with poor chemotherapy response and unfavorable prognosis in breast cancer. We found that CDCA7 expression in breast cancer DTP cells was higher than in non-DTP cells. Furthermore, CDCA7 promotes the expression of autophagy-related proteins *ULK1*, *ATG2A*, and *ATG3* through transcriptional regulation, thereby enhancing the autophagy level in breast cancer DTP cells. Collectively, these changes increased the survival and malignant progression of breast cancer DTP cells under chemotherapeutic stress and represented a potential therapeutic target for breast cancer resistance.

## Materials and methods

### Cell culture

The human breast cancer cell lines T47D, MCF-7, MDA-MB-231, and SUM159 and the mouse 4T1 breast cancer cell line were purchased from the National Collection of Authenticated Cell Cultures (Procell Life Science Technology Co., Ltd.), and authenticated through profiling of short tandem repeats. T47D, MCF-7, SUM159, and 4T1 cells were cultured in DMEM (Gibco, USA) supplemented with 10% fetal bovine serum (FBS) (Gibco), penicillin (100 U/mL, Gibco), and streptomycin (100 μg/mL, Gibco). MDA-MB-231 cells were cultured in Leibovitz’s L-15 medium containing 10% FBS, penicillin (100 U/mL, Gibco), and streptomycin (100 μg/mL, Gibco). Cells were incubated in a humidified atmospheric chamber containing 5% CO_2_ at 37 °C and checked regularly for mycoplasma infection, except for the MDA-MB-231 cells, which were incubated in 100% air.

### Extraction of primary breast cancer

After obtaining ethical approval (KY2021061), tumor tissues from breast cancer patients undergoing chemotherapy were collected to extract primary tumor cells. The specific methods were carried out according to the instructions provided with the commercially available kits (human Tumor Dissociation Kit, Miltenyi Biotec). After preparing a single-cell suspension from the tumor tissues, tumor cells were collected from the intermediate layer using 70/30% Percoll density gradient centrifugation for subsequent experiments. Patients’ information is shown in **Supplementary Table 1**.

### *In vivo* tumorigenicity experiment

To assess the impact of CDCA7 on tumorigenicity *in vivo*, a syngeneic mouse model was established using 4T1-LUC breast cancer cells. Six-week-old female BALB/c mice were randomly allocated into four groups: a CDCA7 overexpression group, a CDCA7 knockdown group, and two corresponding empty vector control groups. Each mouse received an orthotopic injection of 1 × 10^6^ lentivirally transduced 4T1-LUC cells, which stably express firefly luciferase, into the second pair of mammary fat pads. Tumor growth was monitored non-invasively using an *in vivo* bioluminescence imaging system. On days 5, 10, and 20 post-inoculation, mice were intraperitoneally injected with D-luciferin (150 mg/kg). Bioluminescence signals were acquired with an IVIS^®^ imaging system 10 minutes after injection, and total flux (photons/second) within the tumor region was quantified as a measure of tumor burden.

### Flow cytometry analysis of DTP cells

DTP cells are identified in tumor tissues of breast cancer patients undergoing chemotherapy based on their phenotypic characteristics of neither undergoing apoptosis nor proliferating (11). Proliferating cells were marked with Ki67, and apoptotic cells were marked with Annexin V. Cells that are double negative for Ki67 and Annexin V are DTP cells, whereas all others are non-DTP cells. The antibody information used for flow cytometry analysis is shown in **Supplementary Table 2**.

### DTP state induction *in vitro*

DTP variants resistant to Docetaxel (Doc, HY-B0011, MCE) were generated in T47D, MCF-7, MDA-MB-231, SUM159, and 4T1-Luc cell lines through prolonged exposure to cytotoxic concentrations of Doc for specified durations. The emergence of the DTP state was confirmed by two key phenotypic markers: significant increase in drug resistance (as measured by elevated IC_50_ values) and characteristic cell cycle arrest (demonstrated by EdU/PI dual staining assays) (25).

### Cytotoxicity assay

Cytotoxicity was evaluated using cell counting kit-8 (CCK8) assays (ck04, Dojindo). Cells were seeded at a density of 0.5 × 10^4^ cells/well in 200 μL of growth medium in 96-well plates and grown overnight. Cells were then subjected to different treatments. After 72 h incubation, the media were replaced with fresh culture media containing CCK8 solution, and cells were incubated for an additional 4 h at 37 °C. The absorbance was measured at 450 nm using a microplate reader.

### Bioinformatics analysis

Gene Ontology (GO) and the Kyoto Encyclopedia of Genes and Genomes (KEGG) pathway analysis were employed to annotate the biological functions of differentially expressed genes (DEGs) by the R language package (GO plot, KEGG plot function R). Hallmarks of breast cancer chemoresistance were investigated using GSEA.

### Online dataset

Gene expression correlation analysis was performed using the GEPIA2 website (http://gepia2.cancer-pku.cn/#analysis). CDCA7 expression levels in normal breast tissue and tumors of all breast cancer subtypes based on The Cancer Genome Atlas (TCGA) database were analyzed using the ICO website (http://bcgenex.ico.unicancer.fr/BC-GEM/GEM-requete.php). The relative expression of CDCA7 and prognosis based on the TCGA and Gene Expression Omnibus databases were analyzed using the Kaplan–Meier plotter website (https://kmplot.com/analysis/index.php?p=service).

### Western blotting

Cells were harvested and lysed in a lysis buffer (87787, Thermo Scientific) supplemented with a protease and phosphatase inhibitor single-use cocktail (78442, Thermo Scientific). Protein concentrations were determined using a bicinchoninic acid (BCA) assay (23227, Pierce) according to the manufacturer’s instructions. Proteins were separated using 8-10% sodium dodecyl sulfate-polyacrylamide gel electrophoresis (SDS-PAGE) and transferred to polyvinylidene fluoride (PVDF) membranes (1620177, Bio-Rad). Membranes were blocked with 10% bovine serum albumin (BSA) in 0.1% Tween in tris-buffered saline (TBST) buffer for 1 h and incubated in primary antibodies at 4  °C overnight, followed by the incubation with HRP-conjugated secondary antibody at room temperature for 1  h. Standard procedures were used for the western blot analysis. The antibody information used for western blotting is shown in **Supplementary Table 2**.

### RT-qPCR

Total RNA was extracted using TRIzol reagent (15596026, Invitrogen), and a cDNA reverse transcription kit (2509737, Invitrogen) was used to synthesize the first-strand DNA according to the manufacturer’s instructions. Briefly, each well contained a reaction volume of 20 μL, and the reaction was carried out using iTaq universal SYBR green supermix (1725124, Biorad) and a CFX96™ real-time PCR detection system (BioRad). Each gene’s mRNA level was expressed relative to the reference gene. Relative expression was calculated using the comparative Ct method (2^-ΔΔCt^). Primers used for RT-qPCR are listed in **Supplementary Table 3**.

### Plasmid construction and transfection

For overexpression, CDCA7 cDNA was inserted into the GV348 lentivirus vector to construct CDCA7 overexpression vectors and an empty vector was used as a negative control (vector). Subsequently, 293T cells were transfected with plasmid DNA to generate titer lentivirus, which was used to infect breast cancer cells to establish stable cell lines. Infected cells were treated with puromycin (1 μg/mL), and puromycin-resistant clones were isolated. Transfection efficiency was verified using western blotting and RT-qPCR.

Moreover, 4T1 cells were infected with luciferase virus HBLV-LUC-PURO (HH20220110CQLWJ-LP01, HanBio Technology) according to the manufacturer’s instructions to facilitate tracing of the *in vivo* 4T1 cell tumor models.

### Transmission electron microscopy

Transmission electron microscopy analysis was used to visualize autophagic vesicles. Briefly, cells were fixed in 0.1% glutaraldehyde. After dehydration, ultrathin sections were cut in a Leica Ultracut microtome (Leica EM UC7). Lead citrate and/or uranyl acetate were used to stain the samples. Autophagic vesicles were analyzed by Philips EM420 electron microscopy.

### mRFP-GFP-LC3 fluorescence analysis

Breast cancer cells were seeded onto a laser confocal petri dish at a concentration of 2.5×10^5^ cells/well and allowed to adhere overnight. Cells were then exposed to monomeric red fluorescent protein-green fluorescent protein-conjugated-LC3 (mRFP-GFP-LC3) adenoviral vectors (HB-AP2100001, HanBio Technology) according to the manufacturer’s instructions for 24 h. The RFP and GFP puncta in the cells were observed using LMS880 confocal microscope (ZEISS) and counted to evaluate the level of autophagy.

### IHC staining

For IHC staining, tissues were deparaffinized and rehydrated; endogenous peroxidase activity was quenched, and tissues were subjected to antigen retrieval, followed by incubation with incubated with primary antibodies at 4  °C overnight. After washing, the sections were incubated with secondary antibodies at room temperature for 30  min and stained with DAB detection kit (ZLI-9019, ZSGB-Bio), followed by counterstaining with hematoxylin (PV-9001, ZSGB-Bio). Negative control slides were incubated with normal goat serum. All slides were assessed by pathologists and scanned using an Olympus VS120 whole-slide scanner with a 20 × objective. Five fields were randomly selected, and the average percentages of positively labeled cells were determined. Quantification of CDCA7 expression was performed by two independent pathologists, and the mean score was calculated. The IHC or H-score was determined by combining the percentage of positively stained tumor cells and the staining intensity, which was graded as follows: 0, no staining; 1, weak staining (light yellow); 2, moderate staining (yellow-brown); and 3, strong staining (brown). The percentage of cells at each staining intensity level was calculated, and an H-score was assigned using the following formula: [1 × (% cells 1+) + 2 × (% cells 2+) + 3 × (% cells 3+)]. The antibody information is presented in **Supplementary Table 2**.

### Chromatin immunoprecipitation-sequencing

ChIP-seq was performed by SeqHealth Co. (Wuhan, China). Briefly, cells were crosslinked with 1% formaldehyde for 10  min at room temperature and then quenched with 125  mM glycine. The cells were treated with cell lysis buffer, and the nuclei were collected and sonicated to fragment chromatin DNA, which was pre-cleared and then immunoprecipitated with anti-FALG (F1804, Sigma) antibodies. The DNA of input and IP were extracted using the phenol-chloroform method. The high-throughput DNA-sequencing (DNA-seq) libraries were prepared using VAHTS Universal DNA Library Prep Kit for Illumina V3 (Catalog No. ND607, Vazyme). The library products corresponding to 200-500 bp were enriched, quantified, and finally sequenced using a Novaseq 6000 sequencer (Illumina) with a PE150 model. Raw sequencing data were first filtered using the Trimmomatic program (version 0.36), low-quality reads were discarded, and the reads contaminated with adapter sequences were trimmed. The clean reads were mapped to the mouse genome using STAR software (version 2.5.3a), whereas RSeQC (version 2.6) was used for reads distribution analysis. The MACS2 software (version 2.1.1) was used for peak calling.

### Dual luciferase reporter assay

The promoter regions of *ULK1*, *ATG2A*, and *ATG3*, along with their respective site-directed mutants, were individually cloned into the GV534 firefly luciferase reporter vector (Shanghai Genechem Co., Ltd.), generating the constructs ULK1-Luc, ATG2A-Luc, and ATG3-Luc, as well as their mutant counterparts. The CV045 Renilla luciferase vector served as an internal control. MDA-MB-231 cells, pre-infected with either a CDCA7 overexpression lentivirus or an empty vector control, were transfected with the aforementioned luciferase reporter constructs. After 24 hours, dual-luciferase activity was quantified using the Dual-Luciferase Reporter Assay System (72050, Promega) following the manufacturer’s protocol. Firefly luciferase signals were normalized to Renilla luciferase activity for data analysis. Primer sequence information for Site-specific deletion is presented in **Supplementary Table 4**.

### RNA interference

CDCA7-siRNA, CHK2-siRNA and their non-silencing scrambled control siRNA were purchased from Shanghai Genechem Co., Ltd. The sequences corresponding to the indicated siRNAs are listed in **Supplementary Table 5**. Cells were cultured in a serum-free medium and transfected with the mixture of siRNA and LipofectamineTM 3000 Transfection Reagent (Invitrogen) according to the manufacturer’s instructions. After incubation at 37  °C for 4  h, fresh culture medium supplemented with 10% FBS was added to the cells, and the appropriate treatment was performed. shRNA and siRNA sequences is presented in **Supplementary Table 5**.

### Site-specific deletion

Site-specific deletion was performed by the PCR-based QuikChange method. Briefly, specific point mutations in the target DNA sequence were introduced by PCR with DNA polymerase (Phanta Flash Master Mix, Vazyme #P510) employed for high-fidelity amplification, the primers used in this experiment are listed in **Supplementary Table 5**. Then the PCR products were digested with the DpnI endonuclease (Thermo Scientific, ER1702) and transformed into chemically competent E. coli stbl3 cells (Beyotime, D1081M). The mutations were confirmed by sequencing. The site-specific deletion was verified by DNA sequencing (26).

### Statistical analysis

Statistical analyses were performed using IBM SPSS Statistics for Mac (version 23.0; IBM, USA) and GraphPad Prism 8 (GraphPad Software, USA). *In vitro* data are presented as the mean ± SEM of three independent experiments. Unpaired, two-sided Student’s t-tests were used to compare cell survival, relative CDCA7 expression, autophagic puncta, cumulative EPI and siCDCA7-FAM release, and tumor volumes and weights between treatments. Gene expression correlations were evaluated using Pearson correlation analysis. The log-rank test was used for Kaplan-Meier survival analyses. The Kolmogorov-Smirnov test was used to calculate the enrichment score, and permutation testing was used to calculate p-values in the GSEA of the mRNA expression profiles of chemoresistant cells. In all cases, *p<0.05, **p<0.01, and ***p<0.001.

### Data availability

The transcriptomic sequencing data generated with DTP cells were uploaded to the GSA database. GSA under accession ID is HRA006069. The transcriptomic sequencing data generated with PDX tumor tissues were uploaded to the GSA database. GSA under accession ID is HRA006070 and HRA008715. The ChIP-seq data have been uploaded to the GSA database. GSA under accession ID is HRA007872. The Whole exome sequencing results data have been uploaded to the GSA database. GSA under accession ID is HRA009663. Additional data generated in this study are available upon request from the corresponding author.

## Results

### CDCA7 is highly expressed in breast cancer DTP cells

**Figure 1:**
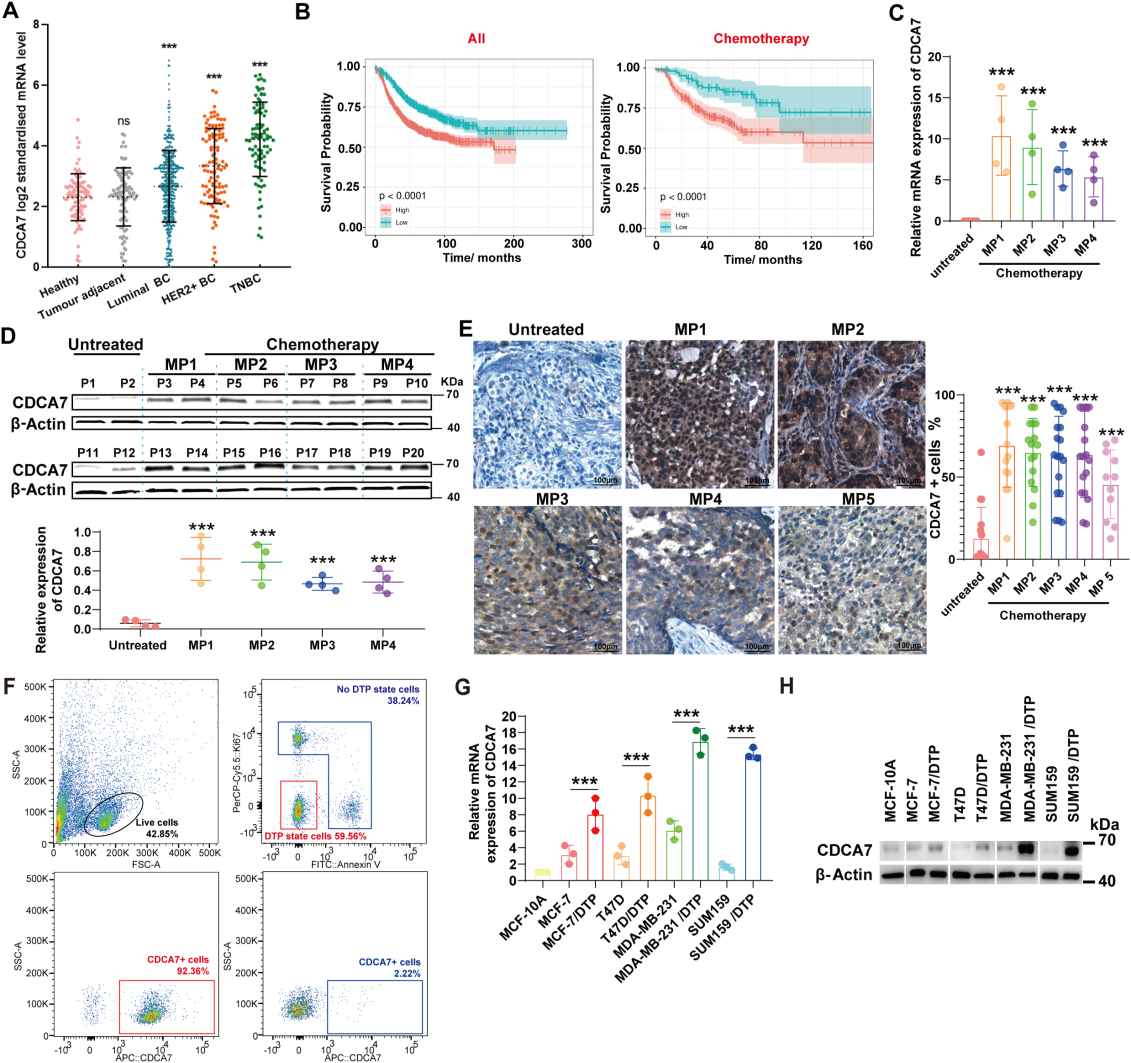
CDCA7 is highly expressed in breast cancer DTP cells. **(A)** CDCA7 has a higher expression in breast cancer tissues than in normal or adjacent non-cancerous tissues based on TCGA database and GTEx database. The data are presented as the mean ± SEM, Healthy:n=92; Tumour adjacent: n=89; Luminal BC: n=524; Luminal BC: n=109;TNBC: n=87. **(B)** Kaplan-Meier curves showing associations between CDCA7 expression levels and survival based on GES16446, GES17907, and GES19615 (high: n=1193; low: n=839, p<0.0001) in all patients with breast cancer, and based on GES16391, GES16446, GES17907, and GES19615 (high: n=259; low: n=107, p<0.0001) in patients with breast cancer who only received chemotherapy, Log-rank test. **(C)** CDCA7 expression levels in tumor cells of breast cancer patients with different chemotherapy response grades. The data are presented as the mean ± SEM, Untreated: n=6; MP1: n=6; MP2: n=6; MP3: n=6; MP4: n=5. **(D)** CDCA7 protein levels in tumor cells of breast cancer patients with different chemotherapy response grades. The data are presented as the mean ± SEM, n=4. **(E)** Immunohistochemical analysis of CDCA7-positive cells in breast cancer tissue. The data are presented as the mean ± SEM, Untreated: n=16; MP1: n=15; MP2: n=16; MP3:n=18; MP4: n=18; MP5: n=11. **(F)** Expression of CDCA7 in DTP and non-DTP cells in breast cancer tissues of chemotherapy patients. **(G)** CDCA7 expression levels in DTP cells of various breast cancer subtypes. **(H)** Protein levels of CDCA7 in DTP cells of various breast cancer subtypes. The data are presented as the mean ± SEM. n=3, Statistical significance was determined by two-tailed unpaired Student’s t-test (two groups) or one-way ANOVA with Dunnett’s multiple comparison tests (multiple groups); ***P < 0.001; ns, no significant difference, unless stated otherwise.

A summary analysis of data from the Cancer Genome Atlas (TCGA) database and the Genotype-Tissue Expression (GTEx) database found that the gene expression level of CDCA7 in breast cancer tissues was higher than in adjacent non-cancerous tissues and normal breast tissues (**Figure 1A**). Chemotherapy is a major treatment for breast cancer, so we further analyzed the relationship between CDCA7 expression and prognosis in breast cancer patients who received chemotherapy. Kaplan-Meier survival analysis showed that among cancer patients receiving chemotherapy, high CDCA7 expression was significantly associated with poor prognosis (**Figure 1B**). By analyzed the CDCA7 mRNA and protein levels in tumor cells of breast cancer patients with different chemotherapy response levels (Miller-Payne grading system), we found that the lower the chemotherapy response, the higher the *CDCA7* expression (**Figures 1C, D**). Similarly, immunohistochemistry assay showed that the number of CDCA7-positive cells was higher in breast cancer tissues with low chemotherapy response compared to those with high chemotherapy response (**Figure 1E**). We used the phenotypic characteristics of DTP cells, which neither undergo apoptosis nor proliferate (Annexin V-Ki67-), to identify DTP cells from breast cancer tissues after chemotherapy, and analyzed the expression of CDCA7 in both DTP and non-DTP cells. The results showed that CDCA7 is mainly highly expressed in DTP cells (**Figure 1F**). We also induced DTP cells in breast cancer cell lines, marked by the appearance of drug resistance and significantly reduced cell proliferation (**Supplementary Figures 1A, B**). In the cell model, we also observed a significant increase in *CDCA7* expression in docetaxel-induced DTP cells (**Figures 1G, H**).

### CDCA7 enhances autophagy in breast cancer cells

**Figure 2:**
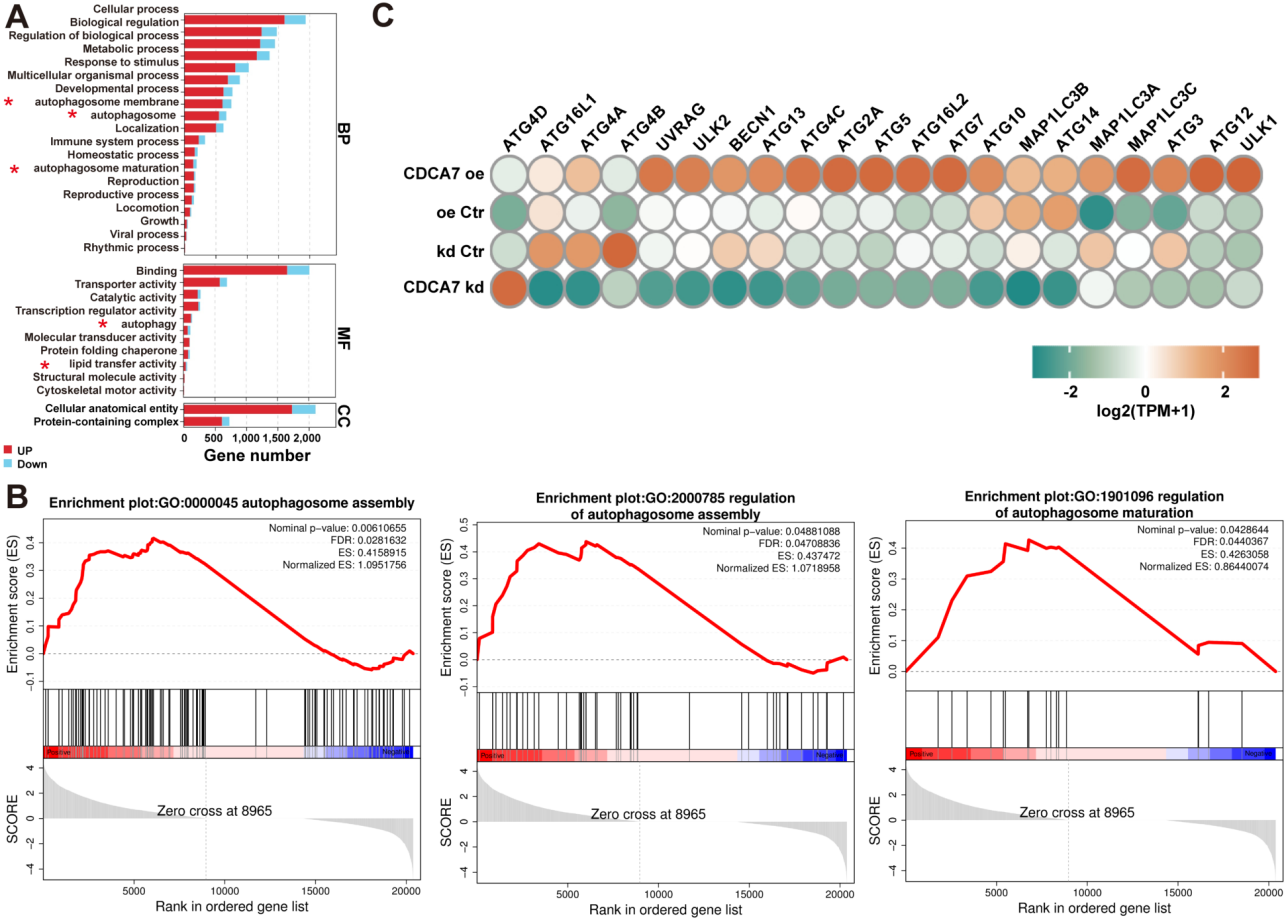
CDCA7 enhances autophagy in breast cancer cells. **(A)** CDCA7 overexpression led to the enrichment of various biological processes and molecular functions related to autophagy activation, based on the GO analysis. Red asterisks indicated autophagy-related biological processes or molecular functions. **(B)** Autophagy-related gene sets, autophagy regulation, and autophagy distribution were significantly enriched in breast cancer cells overexpressing CDCA7, as indicated by GSEA analysis. **(C)** Heatmap of autophagy-related protein expression levels in breast cancer cells (n=3) after interfered with CDCA7 expression.

To further investigate the role of CDCA7 in breast cancer cells, we established breast cancer cell lines with overexpression or knockdown of *CDCA7*, including the human triple-negative breast cancer cell line MDA-MB-231, the luminal breast cancer cell line MCF-7, and the mouse triple-negative breast cancer cell line 4T1. Cells with the optimal level of knockdown were selected for subsequent experiments (**Supplementary Figures 2A–C**). To explore the role of CDCA7 in breast cancer DTP cells, we analyzed the transcriptome of breast cancer cells overexpressing *CDCA7*. Gene Ontology (GO) analysis revealed that *CDCA7* overexpression led to the enrichment of various biological processes and molecular functions related to autophagy activation (**Figure 2A**). Global Gene Set Enrichment Analysis (GSEA) showed that autophagy-related gene sets, autophagy regulation, and autophagy distribution were significantly enriched in breast cancer cells overexpressing *CDCA7* (**Figure 2B**). In addition, we investigated the effect of interfering with *CDCA7* on the expression of autophagy-related genes. We observed that various molecules associated with the autophagy process, such as *ULK1*, *ATG3*, *ATG2A*, *ATG5*, *ATG7*, *UVRAG*, and *BECN1*, were upregulated in *CDCA7*-overexpressing cells and downregulated in *CDCA7*-low-expressing cells (**Figure 2C**). These findings suggest that CDCA7 enhances autophagy levels in breast cancer cells.

**Figure 3:**
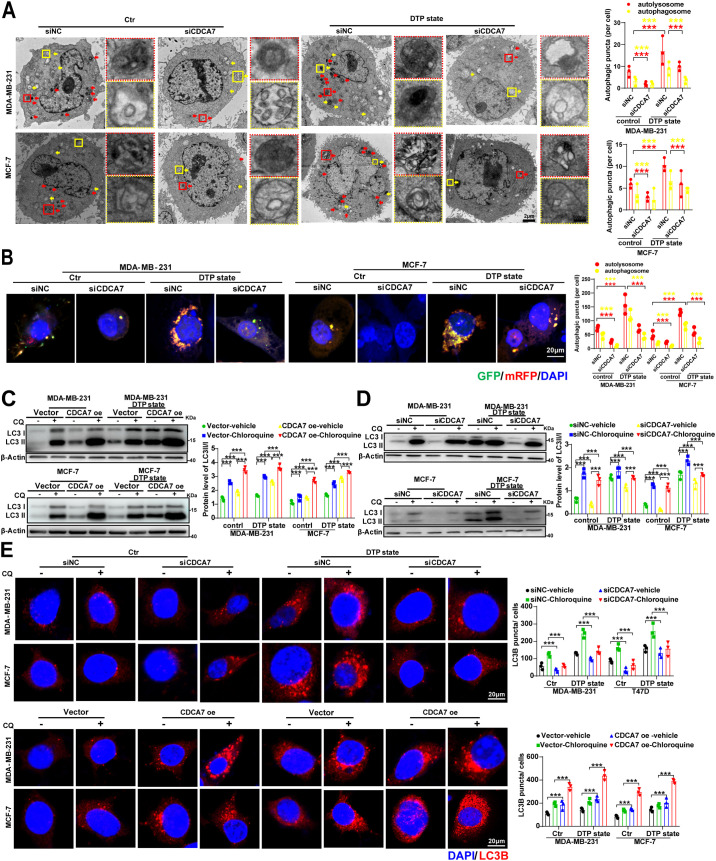
CDCA7 is involved in the autophagy activation of DTP cells. **(A)** Transmission electron micrographs of autophagosomes (yellow arrows) and autolysosomes (red arrows) in DTP cells after the indicated treatment. Scale bar, 20 μm (full field) and 500 nm (insets). **(B)** The abundance of autophagosomes (yellow puncta) and autolysosomes (red puncta) was measured using an LC3-GFP-mRFP reporter in DTP cells after the indicated treatment. Scale bar=20 μm. **(C, D)** Western blot analysis of LC3II/I in MDA-MB-231 and MCF-7 cells with indicated treatments. **(E)** Immunofluorescence analysis and quantification of LC3B staining in MDA-MB-231 and MCF-7 cells. Data are presented as the mean ± SEM. n=3, ***p<0.001; Student’s t-test.

Furthermore, ultrastructural analysis by transmission electron microscopy revealed significantly increased autophagosome and autolysosome accumulation in DTP cells compared to controls, which was markedly attenuated by *CDCA7* knockdown (**Figure 3A**). Confocal microscopy quantification confirmed this observation, demonstrating a reduction in autophagic vesicles upon *CDCA7* silencing (**Figure 3B**). Autophagy flux assays showed that *CDCA7* overexpression enhanced LC3B accumulation. *CDCA7* knockdown suppressed DTP-induced LC3B lipidation under both basal and chloroquine-treated conditions (**Figures 3C, D**). Immunofluorescence analysis further validated these findings, with CDCA7-overexpressing cells exhibiting more LC3B puncta than controls in DTP conditions (**Figure 3E**). Collectively, the above results indicat that CDCA7 as a critical regulator of autophagosome generation and autophagic flux.

### CDCA7 induces chemoresistance by directly upregulating ULK1/ATG2A/ATG3-mediated autophagy in DTP cells

**Figure 4:**
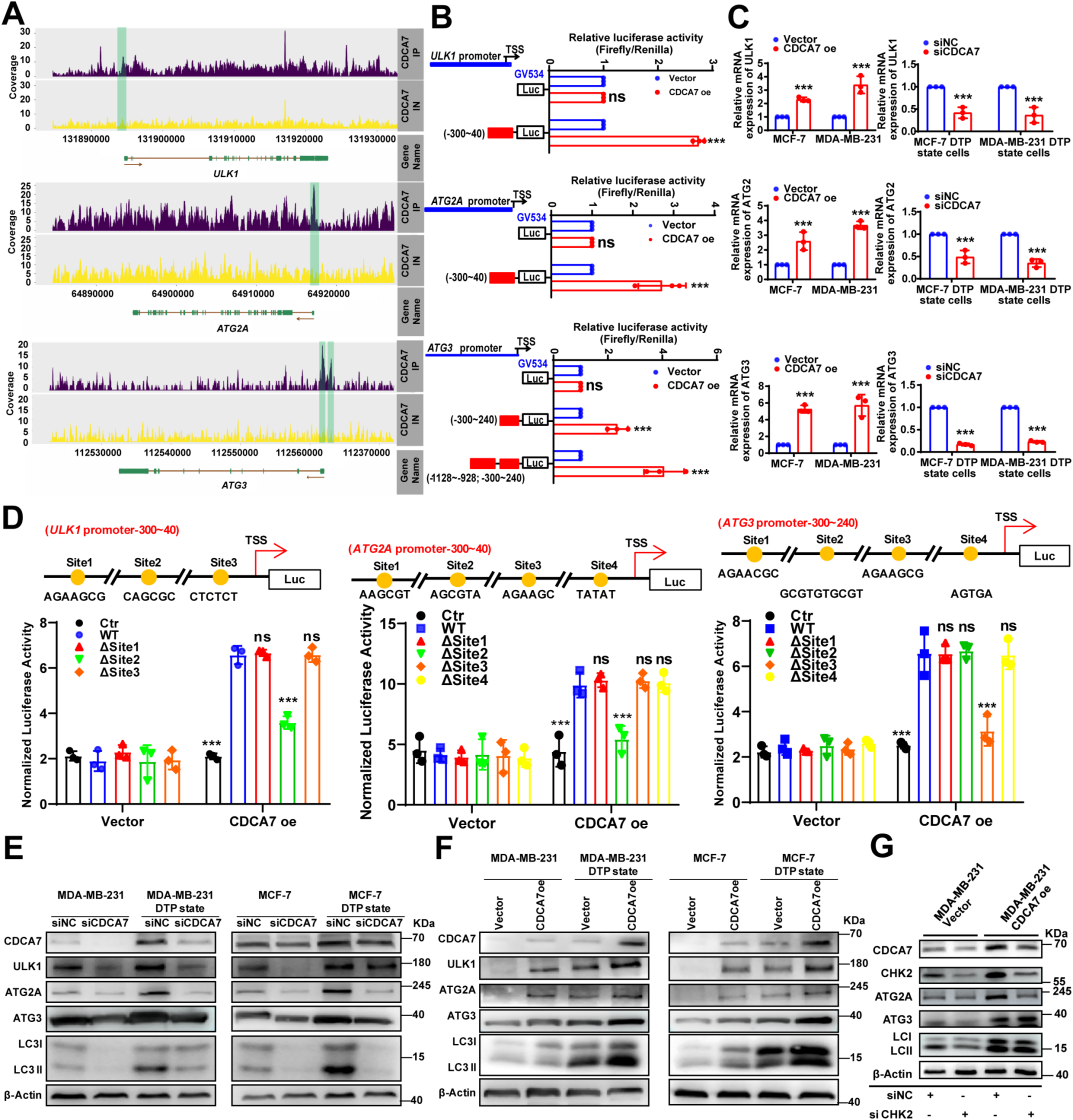
CDCA7 promotes autophagy in DTP cells by upregulating a series of autophagy-related genes. **(A)** Representative images of ChIP-seq peaks against Flag on *ULK1*, *ATG2A*, and *ATG3* promoter in MCF-7 DTP state cells. The green-shaded rectangle indicates the peak site in Chr12:131,894,410-131,894,591 (*ULK1*), Chr11:64,917,110-64,917,277 (*ATG2A*), and Chr3:112,561,723-112,562,005 and 112,562,890-112,563,090 (*ATG3*). **(B)** Luciferase reporter assay of MCF-7 cells using *ULK1* promoter fragment (NM_003565-promoter, -300-40), *ATG2A* promoter (NM_015104.3-promoter, -300-40), and *ATG3* promoter (NM_022488.5-promoter1, -300-240, promoter2, -1128-928, -300-240) after transfection with *CDCA7*-overexpression or empty vector. Data are presented as the mean ± SEM. n=3, ***p<0.001, ns, no significant difference; Student’s t-test. **(C)** Relative expression of *ULK1*, *ATG2A*, and *ATG3* measured using RT-qPCR after *CDCA7* overexpression in breast cancer cells or knockdown in breast cancer DTP state cells. Data are presented as the mean ± SEM. n = 3, ***p<0.001; Student’s t-test. **(D)** Luciferase reporter assay of MCF-7 cells using wild-type *ULK1*, *ATG2A*, and *ATG3* promoter fragments, and their site-directed mutants of each promoter after transfection with *CDCA7* overexpression or empty vector. Relative luciferase activity was normalized to the empty reporter control (GV534). Data are presented as the mean ± SEM. n = 3, ***p<0.001; Student’s t-test. **(E, F)** Western blot analysis of autophagy-related protein expression in breast cancer cells following CDCA7 overexpression or knockdown. **(G)** Western blot analysis of autophagy related protein expression in CDCA7 overexpressing breast cancer cell after treatment with siCHK2.

To investigate the regulatory role of CDCA7 in autophagy-related gene expression, we performed comprehensive analysis of the previous ChIP-seq data. The results revealed significant CDCA7 binding to promoter regions of *ULK1* (Chr12:131,894,410-131,894,591), *ATG2A* (Chr11:64,917,110-64,917,277), and *ATG3* (Chr3:112,561,723-112,562,005 and 112,562,890-112,563,090) (**Figure 4A**). These findings were subsequently validated through dual-luciferase reporter assays targeting each identified binding region (**Figure 4B**). Notably, the ChIP-seq results revealed the presence of two binding sites between the CDCA7 and *ATG3* promoters, with Chr3-112561723–112562005 as the core region. The dual-luciferase reporter gene experiment showed that the regulatory effect of CDCA7 on the *ATG3* promoter was enhanced in both the binding core and binding Chr3-112562890–112563090 regions. We further detected a consistent increase or decrease in the transcription level of *ULK1*, *ATG2A*, and *ATG3* after interfering with CDCA7 expression (**Figure 4C**). Furthermore, based on ChIP-seq analysis, we have identified CDCA7 binding sites within the promoter regions of *ULK1* (Site1: AGAAGCG; Site2: CAGCGC; Site3: CTCTCT), *ATG2A* (Site1: AAGCGT; Site2: AGCGTA; Site3: AGAAGC; Site4: TATAT), and *ATG3* (Site1: AGAACGC; Site2: GCGTGTGCGT; Site3: AGAAGCG; Site4: AGTGA), subsequently validated by deletion analysis. Deletion of site 2 in the *ULK1* and *ATG2A* promoters abolished CDCA7-mediated luciferase activation, whereas site 3 was identified as the functional CDCA7-binding motif in the *ATG3* promoter (**Figure 4D**). These findings demonstrated that CDCA7 transcriptionally upregulated *ULK1*, *ATG2A*, and *ATG3* expression, consequently promoting cellular autophagy activation.

Western blot analysis revealed that CDCA7 knockdown significantly reduced the expression of autophagy-related proteins (ULK1, ATG2A, and ATG3) and the LC3-II/I in DTP-state cells (**Figure 4E**). Conversely, CDCA7 overexpression markedly increased the levels of these proteins and LC3-II/I (**Figure 4F**), confirming CDCA7 as a positive regulator of autophagy flux in breast cancer cells. Given the established role of the CHK2-FOXK axis in autophagy regulation (27), we investigated whether CDCA7 regulated autophagy directly or through CHK2-mediated mechanisms. Western blot analysis demonstrated that CDCA7 overexpression partially rescued the suppression of autophagy-related proteins induced by CHK2 knockdown (**Figure 4G**), indicated that CDCA7 can bypass CHK2 to activate autophagy. Collectively, those results suggest that CDCA7 directly modulates the expression of *ULK1*, *ATG2A*, and *ATG3*.

Based on the critical role of autophagy activation in maintaining the survival of DTP cells, some researchers have also proposed a strategy to inhibit DTP cells through chemotherapy combined with autophagy inhibitors (such as the ULK1 inhibitor SBI-0206965) (28). Here, we also compared which regimen was more effective in killing DTP cells: chemotherapy combined with the autophagy inhibitor SBI-0206965 or combined with siCDCA7. The results indicate that, whether in our induced breast cancer DTP cells or in the screened DTP cells from breast cancer tissues, chemotherapy combined with siCDCA7 consistently demonstrates a superior effect in killing DTP cells (**Supplementary Figures 3A, B**).

### CDCA7 promotes increased multidrug resistance, metastatic invasiveness, and tumorigenicity in breast cancer

**Figure 5:**
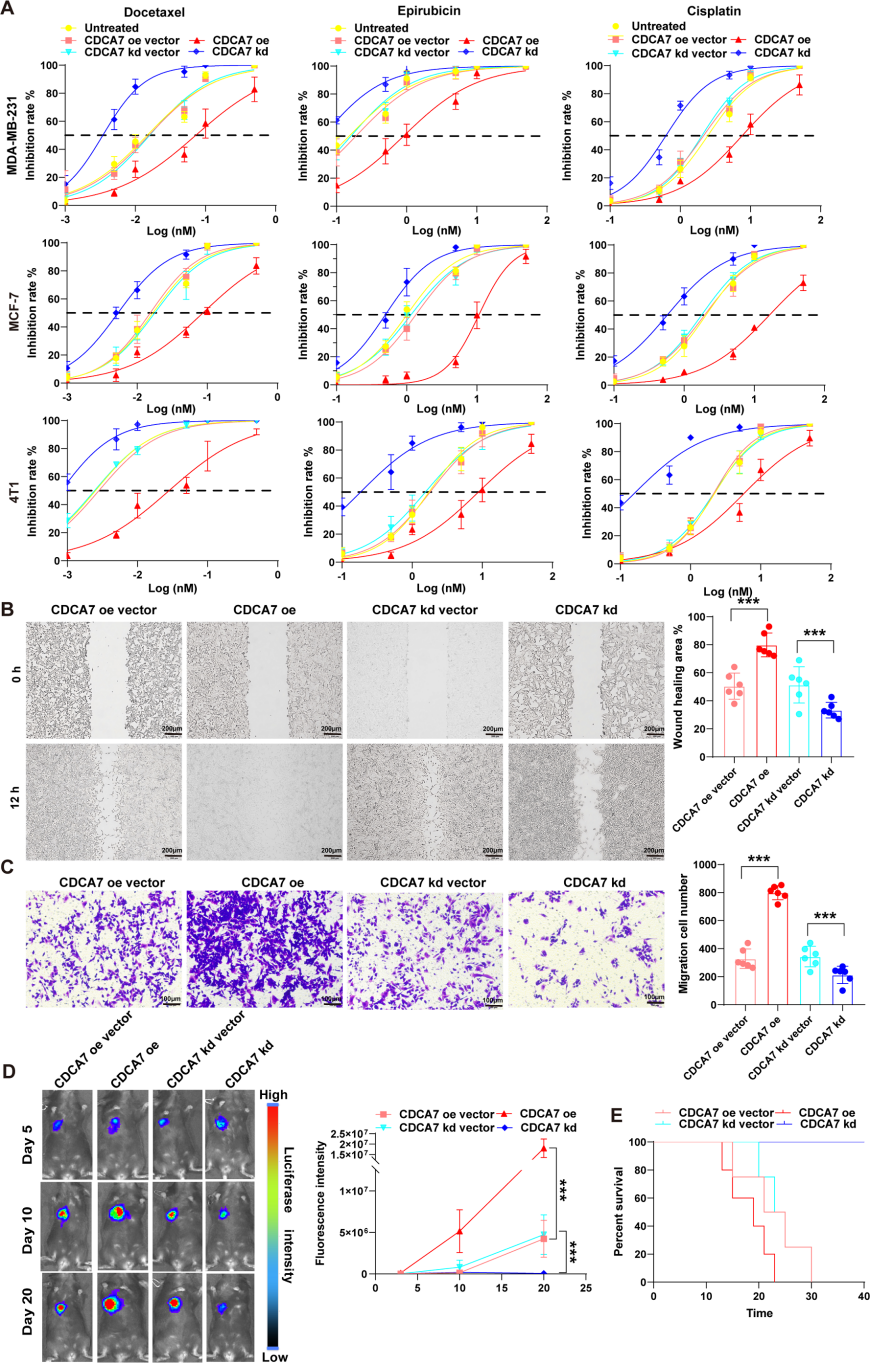
CDCA7 promotes increased multidrug resistance, metastatic invasiveness, and tumorigenicity in breast cancer. **(A)** Assessment of drug resistance in breast cancer cells after interference with CDCA7 expression levels. The IC_50_ value is depicted via a dotted line in the middle of the graph. **(B, C)** Wound healing and Transwell assays were used to detect changes in the migratory and invasive abilities of breast cancer cells following CDCA overexpression or knockdown. Data are presented as the mean ± SEM. n=6, ***p<0.001; Student’s t-test. **(D)** Assessment of the tumorigenic ability of breast cancer cells *in vivo* after interfered with CDCA7 expression. Mean ± SEM. n=5, ***p<0.001; Student’s t-test. **(E)** Survival curve of tumor-bearing mice with breast cancer cells after interfering with CDCA7 expression levels, n=5.

We evaluated the chemosensitivity of breast cancer cells to the first-line chemotherapeutic drugs docetaxel, epirubicin, and cisplatin after interfered with *CDCA7* expression. The results showed that breast cancer cells overexpressing *CDCA7* exhibited increased resistance to all three drugs, while knockdown *CDCA7* enhanced sensitivity to these chemotherapeutic agents (**Figure 5A**). It is noteworthy that *CDCA7* overexpression inhibited the proliferation of breast cancer cells (**Supplementary Figures 4A–C**). To verify the autophagy-mediated mechanism of CDCA7-driven drug resistance, we designed a rescue experiment. We found that the knockdown of *ULK1*, *ATG2A*, and *ATG3* could reverse the drug resistance in breast cancer cells induced by *CDCA7* overexpression (**Supplementary Figure 5**). We further analyzed the effect of *CDCA7* on breast cancer metastasis. The scratch assay showed that overexpression of *CDCA7* enhanced scratch healing and increased metastatic potential. In contrast, *CDCA7* knockdown reduced scratch healing and metastatic activity (**Figure 5B**). In the invasion assay, we observed a significant increase in the number of breast cancer cells overexpressing *CDCA7* that moved through the matrigel to the lower layer, indicating enhanced invasiveness. Conversely, the invasive ability of breast cancer cells with *CDCA7* knockdown was significantly reduced (**Figure 5C**). Finally, we examined the effect of *CDCA7* expression on the tumorigenicity of breast cancer *in vivo*. We observed that, 10 days after implanting equal amounts of tumor cells, the tumor cells in mice with *CDCA7* overexpression were significantly higher than those in the other groups and continued to grow rapidly thereafter. In contrast, the tumorigenicity of the *CDCA7* knockdown group was lower than that of the control group (**Figure 5D**). Ten days after tumor cell implantation, the tumor cells in mice of the *CDCA7* knockdown group gradually decreased, which may be related to the immune response. Accordingly, we also observed that the survival period of mice in the *CDCA7* overexpression group was significantly shortened, while the survival period of mice in the *CDCA7* knockdown group was significantly prolonged (**Figure 5E**).

**Figure 6:**
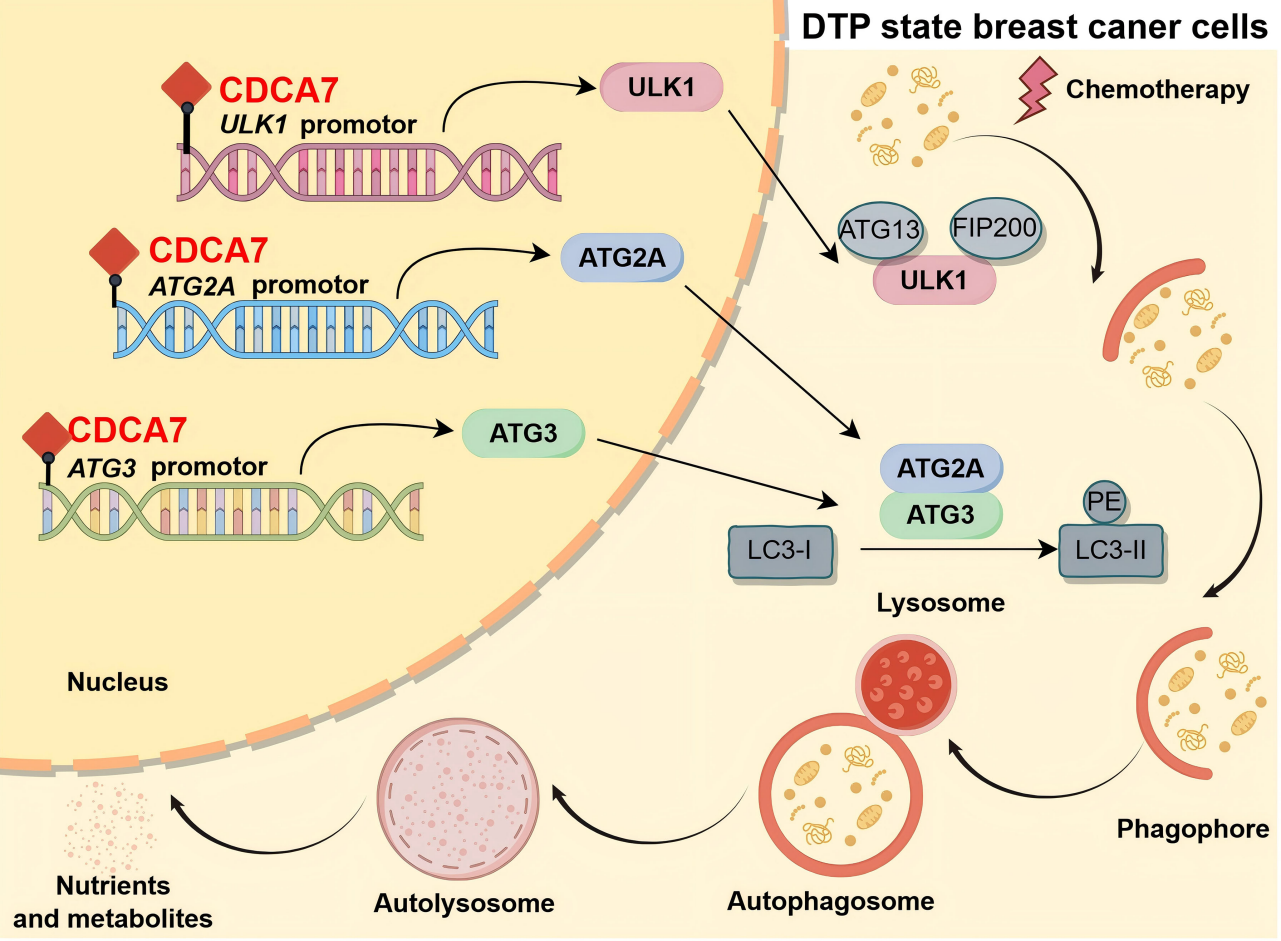
In breast cancer DTP cells, CDCA7 upregulates the expression of *ULK1*, *ATG2A*, and *ATG3* through transcriptional regulation, promoting the early formation of autophagosomes, enhancing the level of autophagy, and facilitating the survival of breast cancer DTP cells.

Therefore, this study investigated the expression levels of CDCA7 in breast cancer DTP cells and its functional significance. Upregulation of CDCA7 expression in breast cancer DTP cells was closely associated with poor clinical outcomes. Furthermore, CDCA7 was associated with autophagy activation in breast cancer DTP cells. Mechanistically, elevated CDCA7 expression promoted transcription of *ULK1*, *ATG2A*, and *ATG3*, thereby facilitated autophagy initiation, phagophore nucleation, and extension (**Figure 6**). Collectively, these changes increased autophagy, ultimately promoting multidrug resistance, invasiveness, and tumorigenicity in breast cancer DTP cells. Based on these findings, we propose that targeting CDCA7 may represent a potential chemotherapeutic sensitization strategy for breast cancer.

## Discussion

Breast cancer is the second most common type of cancer worldwide and ranks fourth in cancer-related mortality (1). Chemotherapy resistance and subsequent recurrence and metastasis are the primary causes of death in breast cancer, making the search for therapeutic targets to overcome resistance a critical issue in its treatment. Previous studies have mainly focused on genetic mutations, such as *TP53*, *PIK3CA*, and *BRCA1/2*, as potential targets to address resistance (29–31). However, the unpredictability of these mutations limits their potential as definitive solutions to tumor resistance (9–11). Therefore, exploring more predictable non-genetic mechanisms has become a promising approach to overcoming resistance.

DTP state refers to a state in which tumor cells commonly, randomly, and non-genetically reversibly enter under chemotherapy or targeted therapy (9–11). It has recently become a new focus in research on tumor drug resistance targets. Currently, there is no consensus on the exact definition of DTP cells. Some researchers characterize DTP cells based on DTP state cells phenotypic features, particularly the coexistence of drug resistance and cell cycle arrest. For instance, DTP cells in tissues can be defined by a phenotype of neither undergoing apoptosis nor proliferating following chemotherapy, whereas *in vitro* cells were defined by a phenotype of drug resistance and proliferative arrest (11, 25). Additionally, certain genes such as *KDM5A*, *ALDH*, *MEX3A*, and *APOBEC3* have been proposed as candidate molecular markers for defining DTP cells (9). In our study, we identified a significantly high expression of CDCA7 in DTP cells of breast cancer cell lines and clinical tumor tissues, suggesting that it could serve as a potential molecular marker for DTP cells in breast cancer. This conclusion requires further validation in a larger set of clinical samples.

The 4CXXC zinc finger domain of CDCA7 can recognize CG-rich DNA methylation sites and function as a transcriptional repressor (32). However, our ChIP-seq data demonstrated that CDCA7 binds to specific promoter motifs (AGAAGCG, CTCTCT, and AGAAGC) and upregulates the expression of *ULK1*, *ATG2A*, and *ATG3* in breast cancer DTP cells. This phenomenon of dual transcriptional regulation - both activation and repression - is typically associated with factors including cellular microenvironment, cell type specificity, binding site selection, recruitment of coregulators, and post-translational modifications (33, 34). Other well-characterized transcription factors exhibiting similar bifunctional regulation include p53, YY1, CTCF, and AP-1 (35–37). Moreover, CDCA7 has been shown to enhance gemcitabine resistance by promoting STAT3 binding to the HK2 promoter, thereby upregulating HK2 expression, while also accelerating cell cycle progression to mitigate iron depletion (38, 39). This evidence collectively indicates that the role of CDCA7 in mediating chemotherapy resistance in DTP cells is not singular; rather, it operates through context-dependent transcriptional programs, reflecting a fundamental mechanism of transcriptional adaptability in response to diverse environmental and therapeutic pressures.

Autophagy is a highly conserved eukaryotic process that is closely related to the progression and resistance of breast cancer (40). However, it is noteworthy that the role of autophagy exhibits essential differences across major breast cancer subtypes (41). HER2-positive breast cancer have been exposed to trastuzumab treatment for a long time develop drug resistance by upregulating autophagy activity, thereby protecting themselves from the inhibitory effect of the drug on growth (42). Among the luminal-type breast cancer subtypes, the autophagy activity of chemotherapy-resistant cells is higher than that of drug-sensitive cells. Inhibiting autophagy can restore chemotherapy sensitivity (43). Most strikingly, TNBC displays constitutive high autophagy dependence, which underpins its aggressive phenotype, stemness, and poor prognosis. This context-dependent functional diversity of autophagy underscores the complexity of targeting this pathway (41).

In this study, we employed an *in vitro* chemotherapy-based model to enrich and characterize DTP cells. While this classical approach offers a controllable system for dissecting cell-autonomous mechanisms underlying DTP formation it inherently lacks key components of the *in vivo* tumor microenvironment, which may contribute to drug resistance through multiple pathway (10). Importantly, to enhance the clinical relevance of our findings, we isolated DTP cells from clinical breast cancer tissue samples and observed that CDCA7 expression was significantly higher in DTP cells compared with non-DTP populations. Mechanistically, we demonstrated that CDCA7 promotes the transcription of autophagy-related genes, elevates autophagic activity, and thereby facilitates chemoresistance and other malignant phenotypes in breast cancer DTP cells. These results suggest that targeting CDCA7 could represent a potential therapeutic strategy to overcome chemotherapy resistance.

## Data Availability

The transcriptomic sequencing data generated with DTP cells were uploaded to the GSA database. GSA under accession ID is HRA006069. The transcriptomic sequencing data generated with PDX tumor tissues were uploaded to the GSA database. GSA under accession ID is HRA006070 and HRA008715. The ChIP-seq data have been uploaded to the GSA database. GSA under accession ID is HRA007872. The Whole exome sequencing results data have been uploaded to the GSA database. GSA under accession ID is HRA009663. Additional data generated in this study are available upon request from the corresponding author.
