# Supplementary material for: CDCA7 promotes chemoresistance of drug-tolerant persister cells in breast cancer by upregulating the expression of autophagy-related protein genes
Source: Front Immunol. 2026 Mar 11;17:1782047. doi: 10.3389/fimmu.2026.1782047 (PMC13013324; doi:10.3389/fimmu.2026.1782047)
Supplement: Supplementary file 1 [file SupplementaryFile1.pdf]

Supplementary Materials

This file includes:

Figures S1 to S5

Tables S1 to S5

Supporting Figure 1

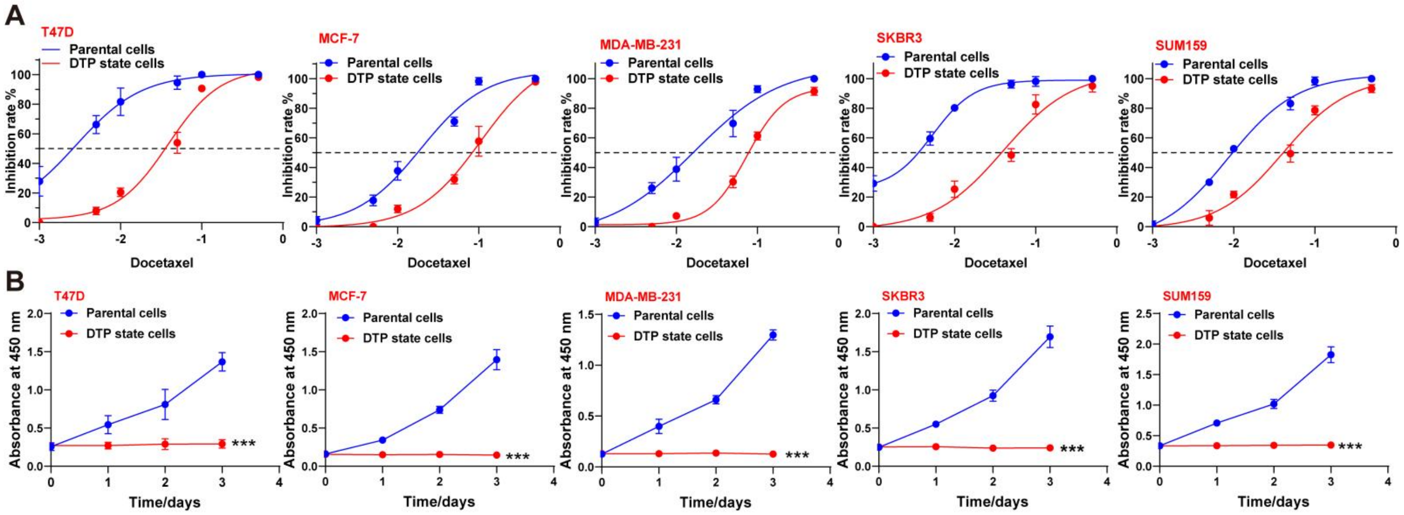

Supporting Figure 1 Drug resistance and cell proliferation arrest serve as endpoint indicators induced by DTP cells. A Assessment of drug resistance in breast cancer cells after interference with CDCA7 expression levels. The IC_{50} value is depicted via a dotted line in the middle of the graph. **B** The curves of cell proliferation. mean ± SEM. n=3, ***p<0.001; Student's t-test.

Supporting Figure 2

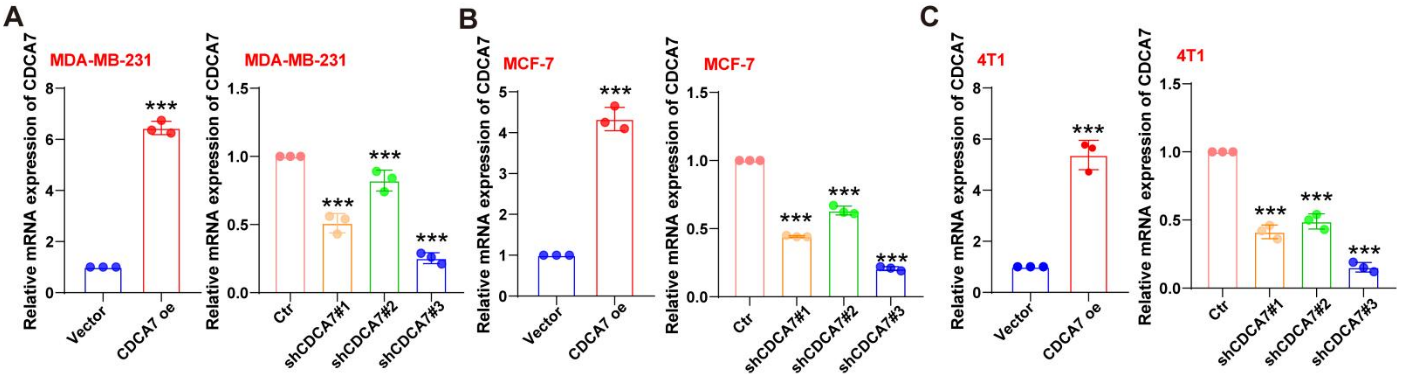

Supporting Figure 2 CDCA7 expression levels in breast cancer cells after infection with a lentivirus overexpressing CDCA7, detected by qRT-PCR (left panel). Knockdown efficiency of three different shCDCA7 lentiviruses, detected by qRT-PCR (right panel). The shCDCA7 #3 construct, which demonstrated optimal knockdown efficiency, was selected for all subsequent mechanistic investigations. Mean ± SEM. n=3, ***p<0.001; Student's t-test.

Supporting Figure 3

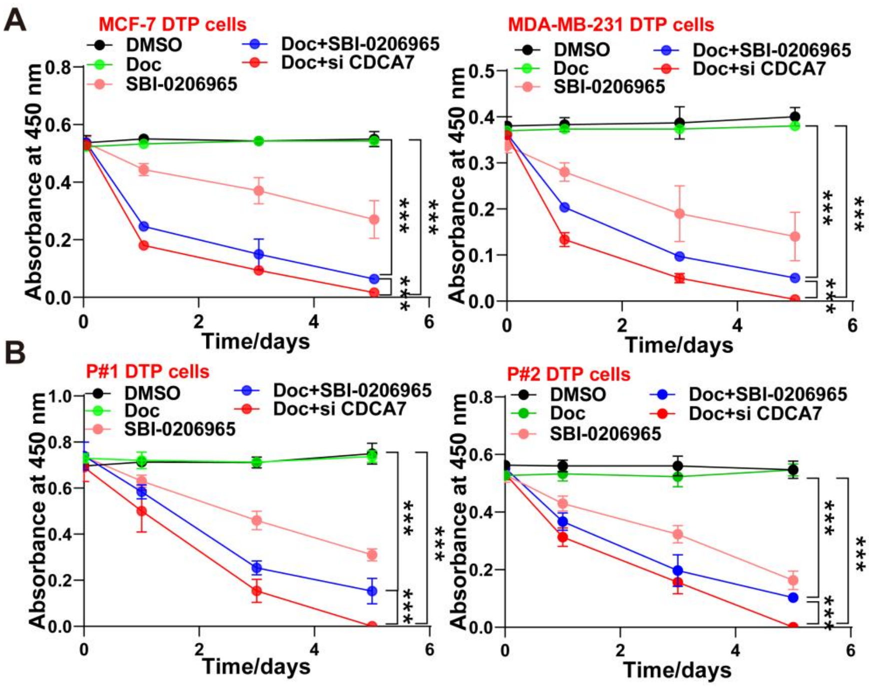

Supporting Figure 3 Inhibitory effect of chemotherapy combined with siCDCA7 on breast cancer DTP cells. A Cell viability of DTP cells induced by breast cancer cell lines after treatment with Doc (0.25 μM), SBI-0206965 (5 μM), and Doc (0.25 μM) combined with siCDCA7 (1 nmol), or SBI-0206965 (5 μM), measured using cell counting kit-8 (CCK8) assay. **B** Cell viability of DTP cells sorted from breast cancer tumor tissue after treatment with Doc (0.25 μM), SBI-0206965 (5 μM), and Doc (0.25 μM) combined with siCDCA7 (1 nmol), or SBI-0206965 (5 μM), measured using cell counting kit-8 (CCK8) assay. Data are presented as the mean ± SEM. n=3, ***p<0.001; Student's t-test.

Supporting Figure 4

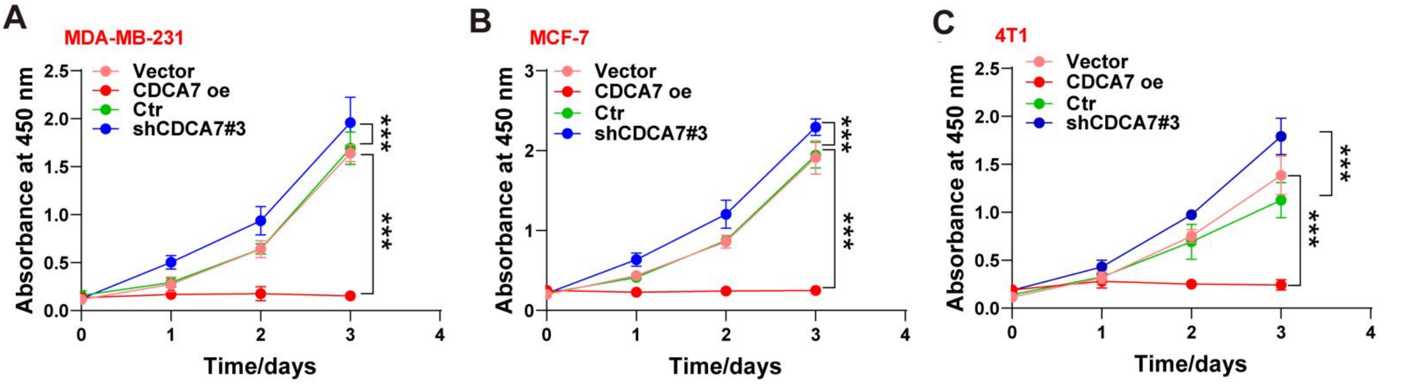

Supporting Figure 4 The curves of cell proliferation. Mean ± SEM. n = 3, ***p<0.001; Student's t-test.

Supporting Figure 5

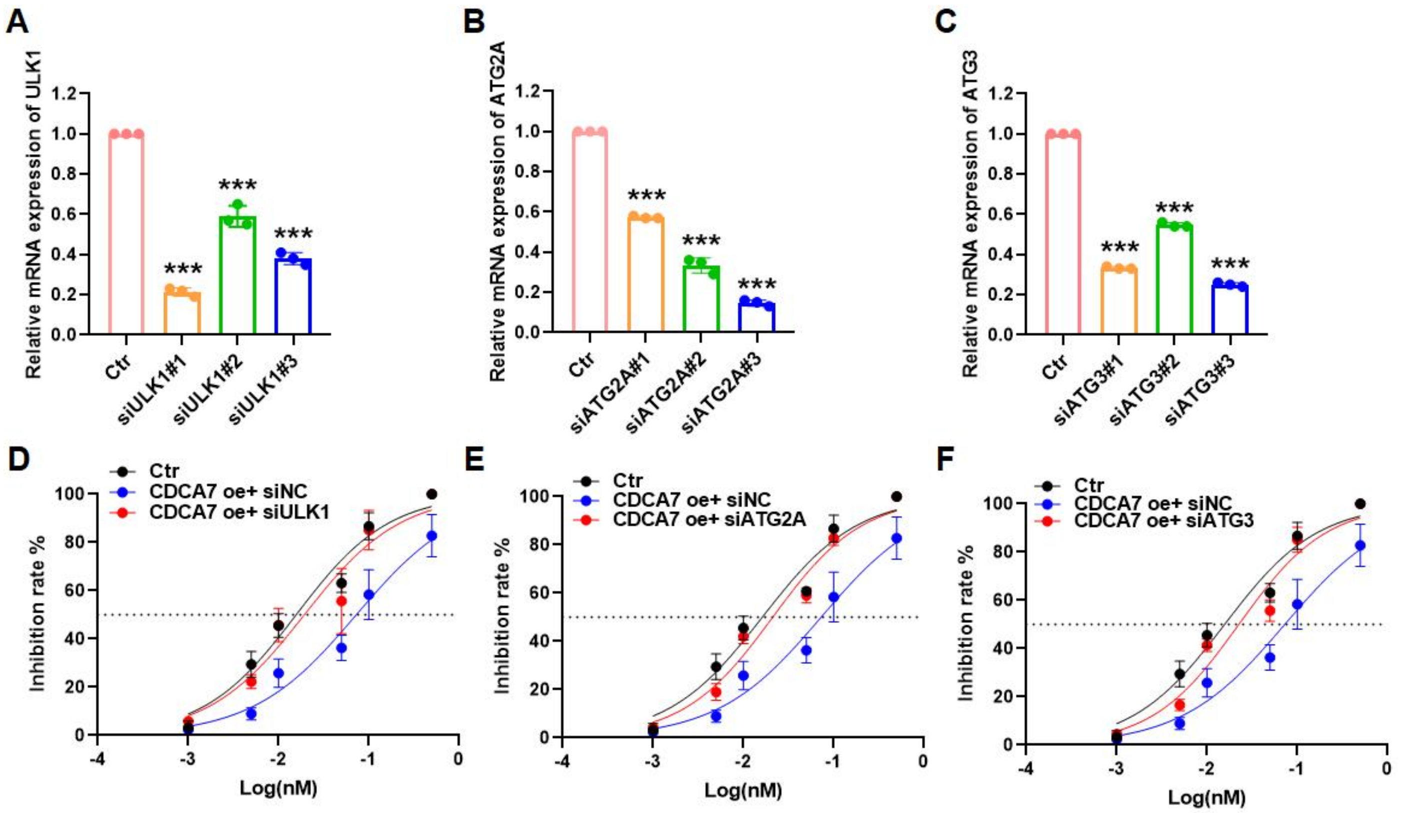

Supporting Figure 5 Down-regulation of autophagy-related genes reverses the drug resistance induced by CDCA7 overexpression. A-C *ULK1*, *ATG2A*, *ATG3* expression levels in MDA-MB-231 cells after knockdown detected by qRT-PCR. **D-F** Assessment of drug resistance in MDA-MB-231 cells after overexpressing *CDCA7* and knockdown *ULK1*, *ATG2A*, *ATG3* expression levels. The IC_{50} value is depicted via a dotted line in the middle of the graph. Data are presented as the mean ± SEM. n=3, ***p<0.001; Student's t-test.

Table S1. Patients' information

Patient	Age (years)	ER (%)	PR (%)	HER2+	Miller-Payne grading
P1	62	80	15	+	Untreated
P2	59	90	50	-	Untreated
P3	62	90	40	+	MP1
P4	57	90	30	-	MP1
P5	56	80	60	+	MP2
P6	66	90	20	-	MP2
P7	62	-	-	-	MP3
P8	64	90	30	+	MP3
P9	65	-	-	-	MP4
P10	60	-	-	+	MP4
P11	67	-85	35-	-	Untreated
P12	52	-68	60-	-	Untreated
P13	65	-	-	-	MP1
P14	55	60	20	++	MP1
P15	61	-	-	+	MP2
P16	60	90	65	+	MP2
P17	59	85	20	+	MP3
P18	54	60	40	+	MP3
P19	63	94	25	+	MP4
P20	64	-	-	+	MP4

Abbreviation

ER: Estrogen Receptor

PR: Progestogen Receptor

HER2: Human Epidermal Growth Factor Receptor 2

TNM: Tumor, Node, Metastasis

Table S2. The antibody and inhibitor information used in this study

For immunohistochemistry staining

Antibody	Dilution	Catalog	Manufacturer
Anti-human CDCA7	1:1 000	PAB21100	Abnova

For flow cytometry

Annexin-FITC	1:500	560931	BD Pharmingen
Ki67-PerCP cy5.5	1:500	561284	BD Pharmingen
CDCA7	1:500	PA5-101299	Invitrogen
Alexa Fluor 647	1:1 000	ab150083	Abcam

For western blotting

Antibody	Dilution	Catalog	Manufacturer
Anti-human CDCA7	1:1 000	PAB21100	Abnova
ULK1 (D8H5) Rabbit mAb	1:1 000	8054T	Cell Signaling Technology
Atg2A Antibody	1:1 000	15011S	Cell Signaling Technology
Anti-human ATG3	1:1 000	3415	Cell Signaling Technology
Anti-human LC3	1:1 000	12741	Cell Signaling Technology
Anti-human β-Actin	1:2 000	8457	Cell Signaling Technology
HRP-conjugated anti-Rabbit IgG	1:5 000	7074	Cell Signaling Technology
HRP-conjugated anti-Mouse IgG	1:2 000	bs-0296G-HRP	Biosynthesis Biotechnology
Pre stained protein molecular weight standard (10 to 180 kDa)	-	26616	Thermo Scientific
BiosPMTM Rainbow Protein Marker (10-310KD)	-	PM1310	Bioss antibodies

For inhibition experiment

Small molecule drugs	Concentration	Catalog	Manufacturer
SBI-0206965	5 μM	HY-13946	Med Chem Express
Chloroquine	50 mg	HY-17589A	Med Chem Express
Docetaxel	5nM	HY-B0011	Med Chem Express
Cisplatin	20 μM	HY-17394	Med Chem Express
Epirubicin	5 μM	HY-13624	Med Chem Express

Plasmid

Vector	Name	Elements
Reporter gene plasmid	GV534	MCS-firefly_Luciferase
Internal control plasmid	CV045	TK promoter-Renilla_Luciferase

Gene name	GENE_ID	Genbank	CDS Size
ULK1	8408	NM_003565-promoter（-300~40)	340
ATG2A	23130	NM_015104.3-promoter（-300-40）	340
ATG3	64422	NM_022488.5-promoter-1(-300~240)	540
ATG3	64422	NM_022488.5-promoter-2(-1128~-928,-300~240)	741

Others

Name	size	Catalog	Manufacturer
Flag M2 mouse produce	50 μg	F1804	Sigma-aldrich
Cell Counting Kit	1 000 test	ck04	Dojindo
BeyoClic EdU-488 Cell Proliferation Detection Kit	2 000 test	C0071L	Beyotime
Dual luciferase reporter assay	100 test	E1910	Promega
Human Tumor Dissociation Kit	-	130-095-929	Miltenyi
Percoll	1L	17089109	Cytiva

Table S3. Primer sequence information for RT-qPCR

Target	Forward (5'→3')	Reverse (5'→3')
hCDCA7	GATCTGGGCACCCGCCACCA	TGACTGCAGCCTCGTGTTTGC
hATG2A	GCTCAGGGTACATGGAGCTG	CTCGTGGTCTGTAAGGCTCAC
hATG3	GAGCTGTACAAGTCTAGAGTGA	CGCAGATCCTTGCGGCCGCGT
hULK1	CCACCCAGTTCCAAACACCT	CCAACTTGAGGAGATGGCGT
hβ-Actin	ATCATGTTTGAGACCTTCAACA	CATCTCTTGCTCGAAGTCCA
mCDCA7	GCAGAACAAAGCAATGCTTG	GGGACCTTGACCTGGTAAGA
mβ-Actin	CCTCACTGTCCACCTTCC	GGGTGTAAAACGCAGCTC

Table S4. Primer sequence information for Site-specific deletion

Target	Forward (5'→3')	Reverse (5'→3')
ATG2A-site1	CACACAGTACTT TACGCAAATCAT GATTGAGAG C	GCTCTCAATCATGATTTGCGTAAAGTA CTGTGTG
ATG2A-site2	TTGCAATAGCCAATGACGGG GGCGTGGCTGGGCGT	ACGCCCAGCCACGCCCCCGTCATTGGC TATTGCAA
ATG2A-site3	CGCGGCCTGGCGCCTAGGGC GGGGCGAGCCGGGG	CCCCGGCTCGCCCCGCCCTAGGCGCCA GGCCGCG
ATG2A-site4	ATCATGATTGAGAGCCCCAT GCAGAGAGCCCAGT	ACTGGGCTCTCTGCATGGGGCTCTCAA TCATGAT
ATG3-site1	GCTTCCGGAAGGAGGGGGCT TATCCCGCCCCAGC	GCTGGGGCGGGATAAGCCCCCTCCTTC CGGAAGC
ATG3-site2	AGGGGTGCGTGTGCGTCCAC CTCAGGTCTCCCTT	AAGGGAGACCTGAGGTGGACGCACAC GCACCCCT
ATG3- site3	AAGCCGGAAGGGGCGCGAG GACAGACAGCTCGCA	TGCGAGCTGTCTGTCCTCGCGCCCCTT CCGGCTT
ATG3- site4	CGCAGAGGGCGAGGGGTCC GCTTCTCACCTCAGG	CCTGAGGTGAGAAGCGGACCCCTCGC CCTCTGCG
ULK1-site1	GTGGCCCGCTCCCGGGTGCT CTCACCGGGGCGGG	CCCGCCCCGGTGAGAGCACCCGGGAG CGGGCCAC
ULK1-site2	AGCTCGCTAGCCAGAGAGG GCGGGGAAGGGGCGG	CCGCCCCTTCCCCGCCCTCTCTGGCTA GCGAGCT
ULK1-site3	GTGGCCCGCTCCCGGGTGCT CTCACCGGGGCGGG	CCCGCCCCGGTGAGAGCACCCGGGAG CGGGCCAC

Table S5. shRNA and siRNA sequences

Target	Sequence (5'→3')
Human shCDCA7 #1	CCTCTGATGACAGTTGTGACA
Human shCDCA7 #2	GCGCTTATGTTACAAGTTGTT
Human shCDCA7 #3	GCCTG CCTTCTACTTCTCAAA
Human siCHK2 #1	GAACAGATAAATACCGAACAT
Human siCHK2 #2	ACGATGCCAAACTCCAGCCAG
Human siCHK2 #3	AGCTAAATCATCCTTGCATCA
Mouse shCDCA7 #1	GCAAACACAAGACTGCAGTTG
Mouse shCDCA7 #2	GAAGTTTCCGGCACGAAATAC
Mouse shCDCA7 #3	GCAAACGATTCCCACTCTGAC
